# Regulation of Dystroglycan Gene Expression in Early Myoblast Differentiation

**DOI:** 10.3389/fcell.2022.818701

**Published:** 2022-03-07

**Authors:** Munerah Hamed, Jihong Chen, Qiao Li

**Affiliations:** ^1^ Department of Cellular and Molecular Medicine Faculty of Medicine, University of Ottawa, Ottawa, ON, Canada; ^2^ Department of Pathology and Laboratory Medicine, Faculty of Medicine, University of Ottawa, Ottawa, ON, Canada

**Keywords:** gene regulation, histone acetylation, histone acetyltransferase, nuclear receptor, dystroglycan, dystrophies, myogenesis

## Abstract

Dystroglycan, a component of the dystrophin-associated glycoprotein complex, connects the extracellular matrix and cytoskeleton to maintain muscle membrane integrity. As such, abnormalities of dystroglycan are linked to different types of muscular dystrophies. In an effort to develop therapeutic approaches to re-establish signal integration for muscle repair and homeostasis, we have previously determined that a clinically approved agonist of retinoid X receptor enhances myoblast differentiation through direct regulation of gene expression of the muscle master regulator MyoD. Using comprehensive omics and molecular analyses, we found that dystroglycan gene expression is responsive to retinoid X receptor-selective signaling in early myoblast differentiation. In addition, the dystroglycan gene is a MyoD target, and residue-specific histone acetylation coincides with the occupancy of histone acetyltransferase p300 at the MyoD binding sites. Consequently, the p300 function is important for rexinoid-augmented dystroglycan gene expression. Finally, dystroglycan plays a role in myoblast differentiation. Our study sheds new light on dystroglycan regulation and function in myoblast differentiation and presents a potential avenue for re-establishing signal integration of a specific chromatin state pharmacologically to overcome muscle pathology and identify additional myogenic interactions for therapeutic applications.

## Introduction

Skeletal muscle is formed by striated myofibers surrounded by the sarcolemma harboring dystrophin-associated glycoprotein complex (DGC) (Rahimov and Kunkel, 2013). Loss of function mutation of the dystrophin gene results in Duchenne Muscular Dystrophy (DMD), underscoring the importance of DGC function in muscle physiology (Serrano et al., 2011; Guiraud et al., 2015). The DGC is a multimeric protein complex, including dystrophin, dystroglycan, sarcoglycans, sarcospan, dystrobrevin, and syntrophins, which warrants mechanical support to the sarcolemma by providing a connection between the extracellular matrix and the actin cytoskeleton (Bhat et al., 2018).

Dystroglycan is a core component of the DGC and comprises the α and β subunits generated from posttranslational cleavage of a single mRNA species encoded by the *Dag1* gene (Ibraghimov-Beskrovnaya et al., 1992). The α unit functions as an extracellular matrix receptor and plays a role in signal transduction (Ervasti et al., 1990; Kostrominova and Tanzer, 1995; Montanaro et al., 1999; Sciandra et al., 2013), whereas the β unit acts as a scaffold for proteins such as Gbr2 and ERK, with its cytosolic domain anchored to actin through interaction with dystrophin (Suzuki et al., 1994; Jung et al., 1995; Rosa et al., 1996; Cavaldesi et al., 2001; Spence et al., 2004).

Abnormal glycosylation of dystroglycan has been shown to associate with numerous muscular dystrophies such as Fukuyama congenital muscular dystrophy, Walker–Warburg syndrome, muscle–eye–brain disease, congenital muscular dystrophy types 1C and 1D, and autosomal recessive limb-girdle muscular dystrophy type 2 (Kobayashi et al., 1998; Brockington et al., 2001a, 2001b; Longman et al., 2003; Toda et al., 2003; Barresi and Campbell, 2006; Hara et al., 2011; Gao and McNally, 2015). Understanding the molecular mechanism of *Dag1* regulation during myogenic differentiation is thus critical for developing strategies to re-establish signal transduction for muscle repair in the clinic.

During cellular differentiation and proliferation, the transcriptional coactivator p300 facilitates a myriad of gene expression in part by acting as a histone acetyltransferase (HAT) (Ogryzko et al., 1996; Chen and Li, 2011). Depletion of p300 significantly reduces the levels of H3K18 and H3K27 acetylation, which are the hallmarks of active transcription in response to nuclear receptor signaling (Jin et al., 2011). Strong evidence from gene deletion studies shows that p300 is particularly required for nuclear receptor signaling and skeletal muscle development (Yao et al., 1998). In addition, the HAT activity of p300 is essential for Myf5 and MyoD gene expression during myogenic commitment and differentiation (Roth et al., 2003; Francetic et al., 2012; Hamed et al., 2013).

Many diseases and conditions, including muscular dystrophies, cancer, and aging, result in muscle atrophy which can be extremely debilitating, but existing treatment option is limited and palliative in nature. The pressing issue is to develop pharmacotherapeutic approaches to prevent and treat muscle-related diseases. In an attempt to discern the molecular basis of myogenic differentiation, we have found that bexarotene, a clinically approved retinoid X receptor (RXR) agonist (Hanish et al., 2018), enhances the differentiation and fusion of myoblasts through the function of RXR as a transcription factor (AlSudais et al., 2016). Mechanistically, RXR directly regulates the expression of muscle-related genes, including Akt2 and muscle master regulator MyoD (Hamed et al., 2017).

The contribution of transcription factors to gene expression depends on the accessibility of their genomic DNA binding sites, which is generally associated with a specific histone modification pattern (Orkin and Hochedlinger, 2011). Many omics analyses have identified p300 occupancy as the best chromatin signature of gene enhancers, and it has since become a valuable perception to identify novel regulatory elements in the study of transcriptional control (Guenther et al., 2007; Heintzman et al., 2007; Asp et al., 2011). As such, we have generated a 14-state chromatin state model based on genome-wide co-occurrence of different epigenetic marks in committed myoblasts using a hidden Markov model-based method (Hamed et al., 2017).

In addition, we have associated p300 occupancy and residue-specific histone acetylation with distinct chromatin states at the onset of myoblast differentiation and delineated the concerted action of MyoD and myogenin with p300 in myogenic expression (Khilji et al., 2018, 2020). Comprehensive multi-omics approaches have allowed us to identify additional genetic targets pertinent to early myogenic regulation. In this study, we characterize the regulation of dystroglycan gene expression by p300 and RXR-selective signaling. Our study also reveals a role for dystroglycan in early myoblast differentiation.

## Materials and Methods

### Mice and Animal Care

C57BL/6J mice (6 weeks old) were obtained from the Jackson Laboratory, housed in a controlled facility (22°C with 30% relative humidity on a 12 h light/dark cycle), and provided with food and water *ad libitum*. Animals were handled as recommended by the guidelines established by the University of Ottawa Animal Care Service and the Canadian Council on Animal Care.

### Cell Culture and Reagents

Mouse primary myoblasts were isolated and differentiated as previously described (AlSudais et al., 2016). C2C12 myoblasts acquired from the American Type Culture Collection (ATCC) were maintained in growth medium (GM), Dulbecco’s Modified Eagle Medium (DMEM) supplemented with 10% fetal bovine serum and 1% penicillin/streptomycin (P/S), at 37°C with 5% CO_2_. For differentiation, GM of 80% confluent C2C12 culture was replaced with differentiation medium (DM), DMEM supplemented with 2% horse serum, for the indicated duration and treatment. Bexarotene (Gniadecki et al., 2007) was purchased from the LC Laboratories, UVI3003 (Nahoum et al., 2007) from the Tocris and puromycin from the Sigma.

### shRNA Knockdown

C2C12 myoblasts were grown in GM to about 30% confluency and transduced at a MOI of 30 with lentiviral particles targeting α-DAG1 in the presence of polybrene (5 μg/ml) following the manufacturer’s protocol (Santa Cruz Biotechnology). Non-silencing shRNA was used as a negative control. Puromycin (2 μg/ml) was used to select pooled stable clones starting 2 days after infecting the myoblasts for a total duration of 3–5 days.

### Reverse Transcription Quantitative PCR Analysis

Total RNA was isolated from the myoblasts using Total RNA kit I (Omega) following the manufacturer’s protocol. Reverse transcription to cDNA was performed using a High Capacity cDNA Reverse Transcription kit (Applied Biosystems). Total RNA was quantified by Nanodrop (ND-1000), and real-time PCR was conducted using a SYBR^®^ Green and HotStarTaq DNA polymerase (Qiagen) on a CFX96 Touch Real-Time PCR Detection System (BioRad). Each sample was PCR amplified in triplicate. Results were analyzed by the threshold cycle (Ct) comparative method using internal controls. Myogenin primers have been previously described (AlSudais et al., 2016), while the *Dag1*, *Rps26*, and *Tbp* primers are designed as follows:

*Dag1*-F: 5′-GGT​TGG​CAT​TCC​AGA​CGG​TA; *Dag1*-R: 5′-CCT​GCT​GCA​GAC​ACC​TTG​AT.

*Rps26*-F: 5′-GCC​ATC​CAT​AGC​AAG​GTT​GT; *Rps26*-R: 5′GCC​TCT​TTA​CAT​GGG​CTT​TG.

*Tbp-*F: 5′, TCATGGA CCAGAACAACAGC; *Tbp*-R: 5′-GCT​GTG​GAG​TAA​GTC​CTG​TGC.

### Western Blot Analysis

Whole-cell extracts were prepared as described previously (Chen et al., 2005). Protein concentration was determined by a Bradford assay (Bio-Rad) and the Multiscan Spectrum Photospectrometer (Thermo). The proteins were separated by SDS-PAGE and transferred to the Immun-Blot PVDF membrane. Following incubation with specific primary and corresponding secondary antibodies, protein bands were detected using SuperSignal West Pico Chemiluminescent Substrate (Pierce Chemicals) and quantified using ImageJ software. Antibodies specific for p300 were obtained from Santa Cruz Biotechnology (sc-584), α-DAG1 from hybridoma IIH6, MyHC from hybridoma MF20, and β-tubulin from hybridoma E7.

### Quantitative Chromatin Immunoprecipitation

C2C12 myoblasts were differentiated for 24 h, cross-linked, and sonicated with a Bioruptor as previously described (Higazi et al., 2011). Equal amounts of chromatin DNA were used for immunoprecipitation with indicated antibodies at 4°C overnight with corresponding normal IgG antiserum as a negative control. Antibodies for p300 and MyoD were obtained from Santa Cruz Biotechnology (sc-584x and sc-32758x). The immunoprecipitants were captured by Dynabeads protein-A, washed sequentially for 20 min with washing buffers A (0.1% SDS, 20 mM Tris-HCl pH 8.0, 150 mM NaCl, 2 mM EDTA, and 1% Triton X-100), buffer B (0.1% SDS, 20 mM Tris-HCl pH 8.0, 500 mM NaCl, 2 mM EDTA, and 1% Triton X-100), buffer C (1% sodium deoxycholate, 20 mM Tris-HCl pH 8, 0.25 M LiCl, 1 mM EDTA, and 1% NP-40), twice for 10 min with buffer TE (10 mM Tris-HCl pH 8.0 and 1 mM EDTA), and then eluted with buffer T_50_E_10_S_1_ (50 mM Tris-HCl, 10 mM EDTA, and 1% SDS) for 30 min at room temperature. Reverse crosslinking was performed at 65°C overnight. The ChIP DNA was purified using the DNA purification kit (Qiagen), and real-time PCR was performed using a SYBR^®^ Green and HotStarTaq DNA polymerase (Qiagen) on a CFX96 or CFX384 Touch Real-Time PCR Detection System (BioRad). Each sample was amplified in triplicate PCRs. Purified input chromatin DNA was used to generate a standard curve for the PCR amplification of each immunoprecipitation. The abundance of immunoprecipitated target DNA was quantified as the percentage of input chromatin DNA. *Dag1* ChIP primers are designed as follows:

Promoter: F5′-CCTTTCCCAACTTCTCCCGAA; R5′-AGATACCGCTTGCGTTTTGC.

Enhancer: F5′-GCTGGATCTTTGTGGGGCAT; R5′-TGAAAGGAGGAGCCATTGGT.

### Immunofluorescence Microscopy

At the indicated time point, C2C12 myoblasts were stained with a myosin heavy chain antibody (MF20 in 1:10 dilution) as previously described (le May et al., 2011). The cells were then washed with 1X PBS and incubated with Alexa Flour^®^594 secondary antibody (Invitrogen) and 0.1 μg/ml of Hoechst (Molecular Probes) to stain the DNA. The coverslips were mounted on slides with 50% glycerol. Axiovert 200M microscope, AxioCam HRM camera, and AxioVision Rel 4.8 software (Zeiss) were used to capture the images through a ×10 objective. For each coverslip, five random images were analyzed. ImageJ software was used for cell counting.

### Statistical Analysis

**FIGURE 2:**
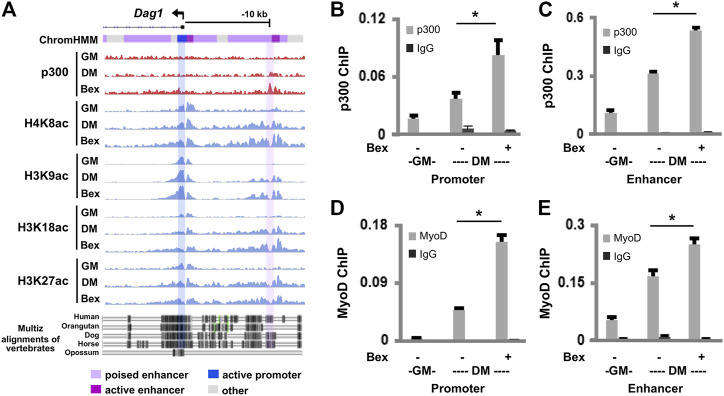
p300 and MyoD occupancy at the *Dag1* locus. **(A)** Genome browser view of p300 and the indicated histone acetylation enrichment signals at the *Dag1* locus. Black bars show Ref-seq gene position and the ChromHMM track coded by the color designated to each chromatin state. The bottom panel displays the conservation of the *Dag1* locus from the alignment of multi-vertebrates using the UCSC genome browser. The C2C12 myoblasts were differentiated for 24 h (DM) in the presence or absence of bexarotene (Bex, 50 nM) with proliferating myoblasts as controls (GM). Quantitative ChIP analysis was performed for the *Dag1* promoter and the putative enhancer, using antibodies specifically against p300 **(B,C)** or MyoD **(D,E)**. Normal IgG antiserum was used as a negative control. Quantification is presented as the percentage of enrichment in relation to the input chromatin DNA (*n* = 3; *p < 0.05).

**FIGURE 3:**
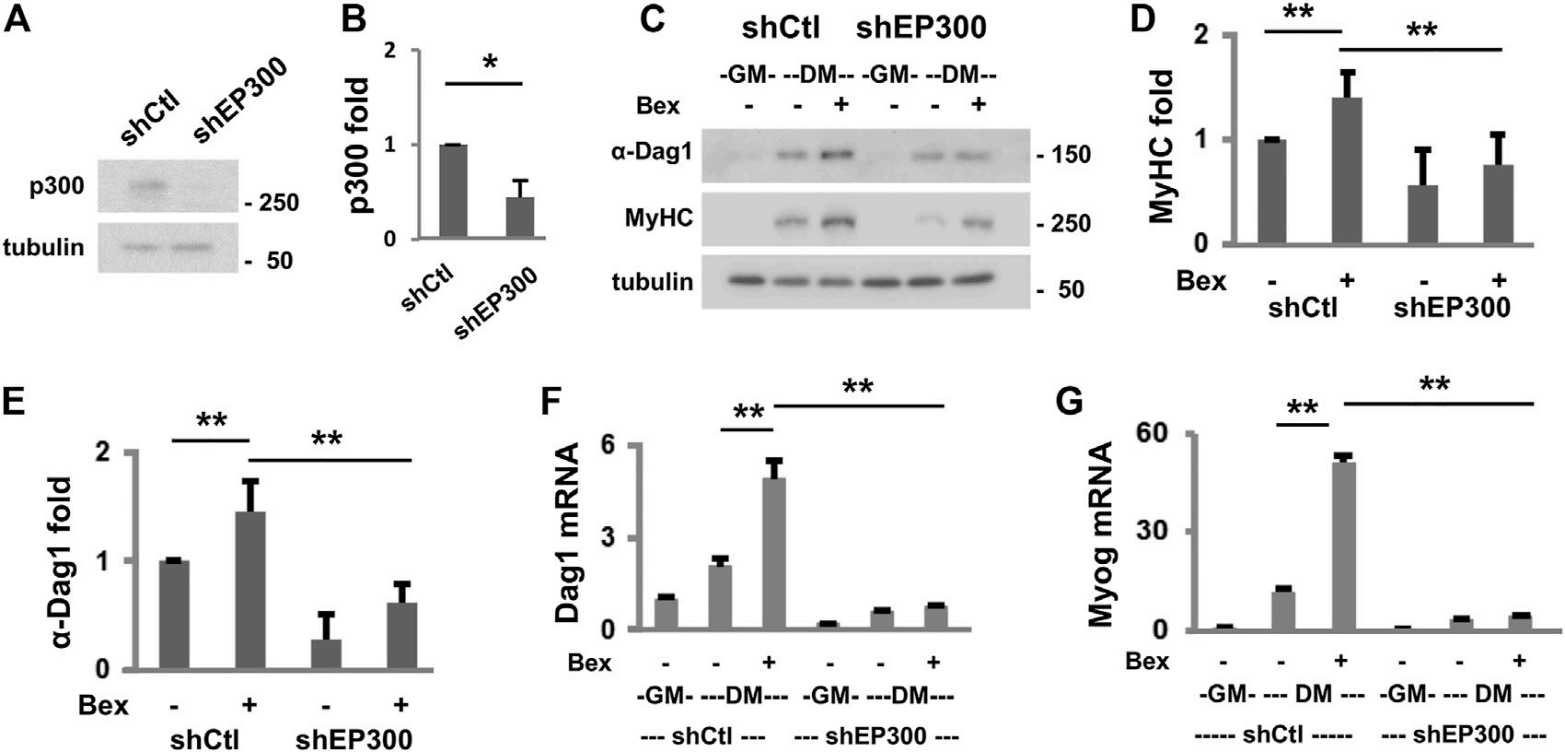
p300 is important for *Dag1* gene expression. **(A)** Endogenous p300 protein in the differentiating myoblasts was analyzed using Western blotting following the introduction of p300 shRNA (shEP300) into the parental C2C12 myoblasts with β-tubulin as loading controls. Non-silencing shRNA (shCtl) was used as a negative control. **(B)** Quantification of the p300 blots is presented as the fold change relative to that of shCtl (*p < 0.05; *n* = 3). **(C)** Western blotting was used to analyze the protein levels of α-DAG1 and MyHC in the p300 knockdown and shCtl myoblasts differentiated in the presence or absence of bexarotene (Bex, 50 nM). Quantification of MyHC **(D)** and α-DAG1 **(E)** protein blots is presented as the fold change in relation to that of untreated differentiating myoblasts (DM, **p < 0.01; *n* = 5). *Dag1* **(F)** and myogenin **(G)** mRNA levels in differentiating shEP300 and shCtl myoblasts were also analyzed by RT-qPCR analysis. Quantification is presented as fold changes relative to proliferating myoblasts (GM, *n* = 3), normalized to *Tbp*.

**FIGURE 4:**
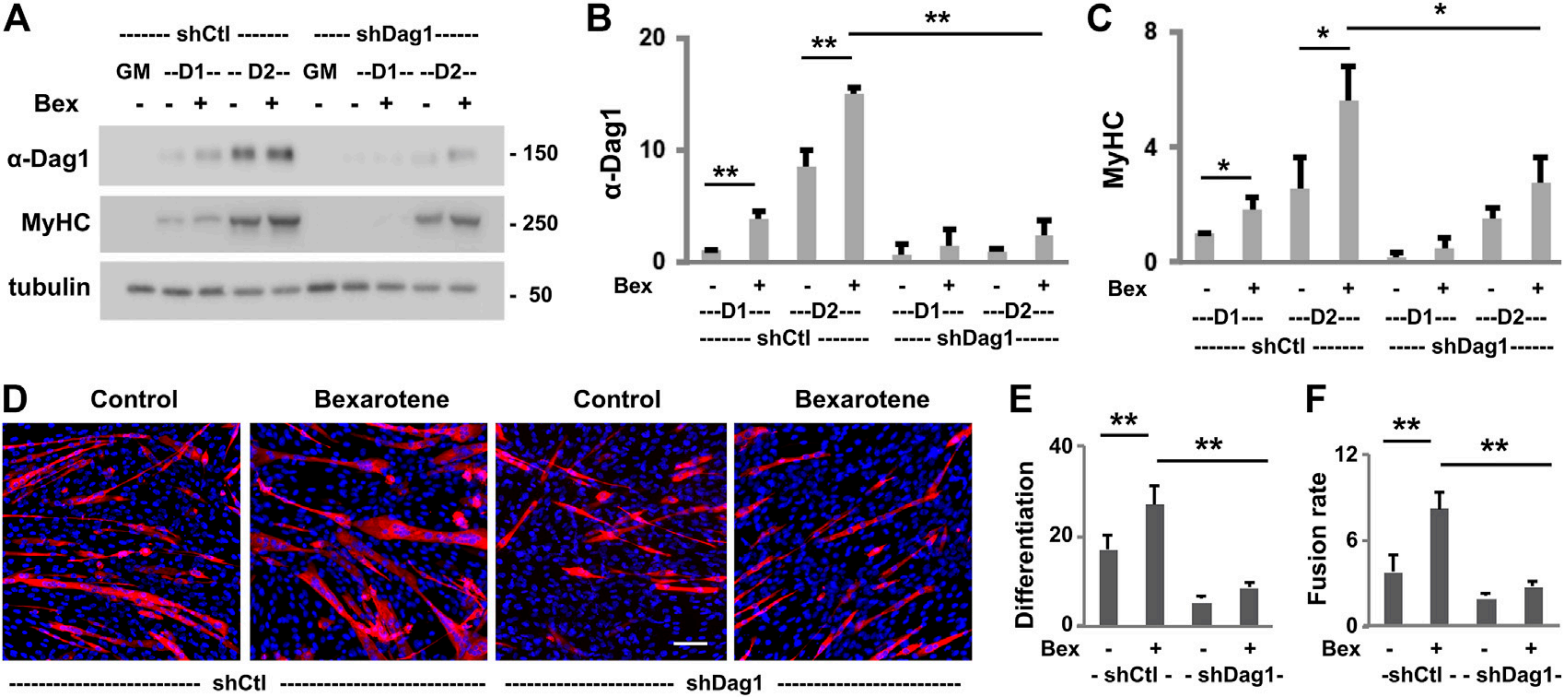
Effects of DAG1 knockdown on myoblast differentiation and fusion. **(A)** C2C12 myoblasts with *Dag1* shRNA knockdown (shDag1) or non-silencing shRNA (shCtl) were differentiated for 1 or 2 days in the presence or absence of bexarotene (Bex, 50 nM) and subjected for Western blotting. Proliferating myoblasts were used as controls (GM). MyHC is included as a myogenic differentiation marker and β-tubulin as a loading control. Quantification of the protein levels of DAG1 **(B)** and MyHC **(C)** is presented as fold change in relation to that of untreated differentiating myoblasts (*p < 0.05; **p < 0.01; *n* = 3). **(D)** shDag1 knockdown and control myoblasts were differentiated for 4 days with or without bexarotene. The representative images (scale bar: 100 μM) stained with MyHC (red) and nuclei (blue). **(E)** Differentiation was defined as the percentage of myogenic nuclei relative to the total number of cell nuclei (*n* = 4). **(F)** Fusion rate was defined as the average number of nuclei per MyHC positive cell.

**FIGURE 1:**
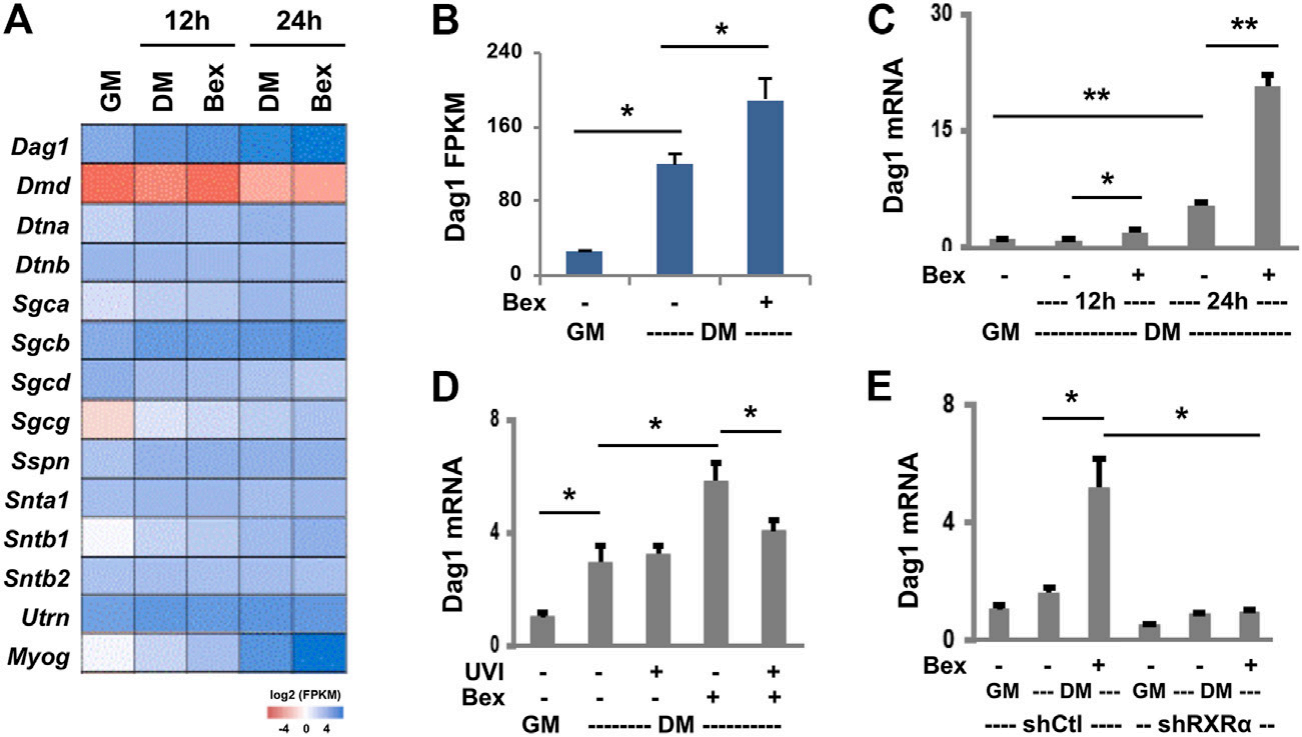
RXR-selective signaling augments *Dag1* expression. **(A)** C2C12 myoblasts were differentiated (DM) in the absence or presence of bexarotene (Bex, 50 nM) for 12 or 24 h and then subjected to RNA-seq analysis in duplicate with proliferating myoblasts as control (GM). Heat map of bexarotene-responsive *Dag1* gene expression with other components of DGC and myogenin in parallel as a comparison. **(B)** Quantification of *Dag1* read signals at 24 h of differentiation is plotted as FPKM (fragments per kilobase of transcript per million mapped reads, *p < 0.05). **(C)** Primary myoblasts were differentiated with bexarotene (Bex, 30 nM). The relative levels of *Dag1* mRNA were determined by RT-qPCR and presented as fold changes in relation to proliferating primary myoblasts, after being normalized to *Rps26* (**p < 0.01; *n* = 3). **(D)** The C2C12 myoblasts were differentiated with bexarotene (50 nM) with or without RXR antagonist UVI3003 (UVI, 1 μM) for 24 h. The relative levels of *Dag1* mRNA were determined by RT-qPCR and presented as fold changes in relation to proliferating myoblasts, normalized to *Rps26* (*n* = 3). **(E)** shRXRα knockdown C2C12 myoblasts were differentiated for 24 h and subjected to RT-qPCR analysis. The relative levels of *Dag1* mRNA were presented as fold changes in relation to proliferating myoblasts, normalized to *Tbp* (*n* = 3).

Statistical analyses were performed using a two-tailed Student’s *t*-test, and *p* < 0.05 was considered statistically significant. Data are presented as the mean ± STDEV, except for RT-qPCR and qChIP, which are presented as the mean ± SEM. Each experiment was repeated at least three times. Statistical details and the number of replicates are indicated in the legends for Figures 1–4.

## Results

### Impact of RXR Signaling on Dystroglycan Gene Expression in Early Myoblast Differentiation

The role of dystroglycan is known for connecting the extracellular matrix and cytoskeleton to maintain skeletal muscle integrity (Barresi and Campbell, 2006). Nevertheless, our recent RNA-seq datasets (Hamed et al., 2017) on a well-characterized C2C12 myoblast model (Webster et al., 1988) showed that *Dag1* gene expression is upregulated in early myoblast differentiation and further augmented by RXR-selective signaling (Figures 1A,B). To determine the impact of RXR signaling on *Dag1* gene expression, we sort to examine in detail the levels of *Dag1* expression in different models of rexinoid-augmented myogenic expression.

First, we used primary murine myoblasts for the study. The primary myoblasts were isolated from the lower hind limb muscles (Megeney et al., 1996) and induced to differentiate in the presence or absence of an RXR-selective ligand, bexarotene, at a concentration of close to the *K*_*d*_ value (30 nM), an optimal treatment condition determined through the titration experimentation. As shown in Figure 1C, upon 1 day of differentiation, there was a fivefold upregulation of *Dag1* mRNA relative to undifferentiated primary myoblasts, and the increased *Dag1* mRNA was further significantly augmented by an additional threefold upregulation following treatment with bexarotene compared with untreated myoblasts. The ability of bexarotene to augment *Dag1* gene expression in primary myoblast differentiation signifies a physiological relevance of rexinoids in the regulation of *Dag1* gene expression.

For molecular dissecting, we next employed the C2C12 myoblasts because they are less prone to spontaneous differentiation and amenable to genetic manipulation and the resulting stable clones retain their capacity to differentiate. First, we employed a potent RXR antagonist UVI3003 (Nahoum et al., 2007) to determine whether the positive effect of rexinoids on *Dag1* gene expression is specifically mediated through RXR activation. The C2C12 myoblasts were differentiated with bexarotene at 50 nM, close to the *K*_*d*_ value, in the presence of UVI3003 at a 30-fold concentration of the *K*_*d*_ value (AlSudais et al., 2016). As shown in Figure 1D, there was a threefold increase in the level of *Dag1* mRNA by 1 day of differentiation, further augmented by the addition of bexarotene, as determined by RT-qPCR. However, cotreatment with the RXR antagonist attenuated the positive effects of bexarotene on *Dag1* mRNA expression, whereas treatment with UVI3003 in the absence of bexarotene did not affect normal *Dag1* gene expression (Figure 1D). Together, our data suggest that bexarotene augments *Dag1* gene expression through RXR-selective signaling.

RXRα is the predominant RXR subtype expressed in skeletal muscle (Barbosa-Morais et al., 2012), and we have previously shown that the RXRα subtype is essential for rexinoid-enhanced myogenic differentiation (AlSudais et al., 2016). We thus delineated further the role of RXR in regulating *Dag1* gene expression using our established pooled stable shRXRα and non-silencing shRNA myoblasts as control. The RXRα knockdown and control myoblasts were differentiated in the presence or absence of bexarotene. As shown in Figure 1E, the augmentation of *Dag*1 gene expression by bexarotene was blunted in the shRXRα myoblasts compared with the control, as determined by RT-qPCR analysis. Therefore, the knockdown of RXRα impaired bexarotene-augmented *Dag1* gene expression, and rexinoids augment *Dag1* gene expression through the activation of RXR. Nonetheless, gene expression of *Dag1* in normal differentiation was also negatively impacted by the knockdown of RXRα (Figure 1E), indicating that unliganded RXRα may play a role in myogenic expression as well.

### Regulation of *Dag1* Gene Expression by p300

Because rexinoid-responsive gene expression is mediated through MyoD that is known to interact with HAT p300 (Hamed et al., 2017), we performed a comprehensive analysis of the *Dag1* locus using our recent ChIP-seq datasets of p300 and histone acetylation in combination with our established chromatin state model (Hamed et al., 2017; Khilji et al., 2018, 2020) to examine the involvement of p300 and histone acetylation signature in *Dag1* gene expression in view of RXR signaling. The Genome browser view of ChIP-seq read signals displayed the occupancy of p300 accompanied by the H4K8ac, H3K9ac, H3K18ac, and H3K27ac signal enrichments at the *Dag1* promoter and a region about 10 kb upstream of the transcription start site (TSS), which was denoted as a poised enhancer based on our myoblast chromatin state model (Figure 2A). In addition, this upstream region is highly conserved across vertebrate species, including humans (Figure 2A), suggesting a putative regulatory region of *Dag1*. More importantly, the signal density of p300 and histone acetylation increased in differentiating myoblasts and further augmented following bexarotene treatment (Figure 2A).

Next, we used quantitative ChIP analysis to ascertain the involvement of p300 in the regulation of *Dag1* gene expression. Two sets of primers were designed spanning the summits of p300 peaks at the *Dag1* promoter and the putative enhancer, respectively. As shown in Figures 2B,C, p300 occupancy at the *Dag1* promoter and the putative enhancer was enriched twofold, respectively, following 1 day of differentiation compared to the undifferentiated myoblasts. Moreover, the enrichment of p300 at the *Dag1* promoter and the putative enhancer was further augmented twofold, respectively, upon bexarotene treatment (Figures 2B,C).

MyoD is a master regulator of myogenesis (Tapscott, 2005) and functions in concert with p300 to regulate myogenic expression (Hamed et al., 2017). As we have detected MyoD signal enrichments at the p300 associated *Dag1* locus (Supplementary Figure S1) through a publicly available dataset (Yue et al., 2014), we next examined the binding of MyoD to the *Dag1* promoter and the putative enhancer using qChIP analysis. Similar to p300, the binding of MyoD to the *Dag1* promoter and the putative enhancer also increased upon differentiation and was further augmented by the addition of bexarotene, respectively (Figures 2D,E). Taken together, our data suggest that p300 and MyoD are involved in both normal and rexinoid-augmented *Dag1* gene expression during early myogenic differentiation.

We also sort to determine the requirement of p300 function in *Dag1* gene expression. First, we used our established p300 shRNA knockdown C2C12 myoblasts (Chen et al., 2015) to assess the levels of DAG1 protein in early myogenic differentiation (Figures 3A,B). As shown in Figures 3C,D, the knockdown of the endogenous p300 resulted in a significant reduction in the level of myosin heavy chain, a muscle-specific gene, in differentiating myoblasts as determined by the quantitative Western blot analysis, reflecting the importance of p300 function in myogenesis. Most importantly, the levels of DAG1 protein were also significantly decreased following p300 knockdown in bexarotene treated and untreated differentiating myoblasts, respectively, compared to corresponding non-silencing shRNA control myoblasts (Figures 3C,E). In addition, the negative impact of p300 knockdown on *Dag1* gene expression is exhibited by a reduced level of *Dag1* mRNA in early differentiation, which is similar to that of myogenin, a marker of myoblast differentiation (Singh and Dilworth, 2013), as shown by the quantitative RT-PCR analysis (Figures 3F,G). Taken together, our data suggest that p300 function is important for *Dag1* gene expression, possibly through the regulation of *Dag1* promoter and the putative enhancer in early myogenic differentiation.

### The Role of Dystroglycan in Early Myoblast Differentiation

The involvement of p300 and MyoD in rexinoid-augmented *Dag1* gene expression suggests a functional role of DAG1 in early myoblast differentiation because both p300 and MyoD are critical players in early myogenic differentiation. We, therefore, delineated further the functional requirement of DAG1 in myogenic differentiation. To this end, we targeted DAG1 to generate pooled stable C2C12 myoblasts for a loss of function study. Gene-specific shRNA was used to knock down the endogenous DAG1, and non-silencing shRNA was used as a negative control. Validation experimentation showed that DAG1 protein levels increased continuously during the process of myoblast differentiation and was further augmented by the addition of bexarotene in the shRNA control myoblasts (Figures 4A,B). However, the introduction of the *Dag1* shRNA effectively knocked down the endogenous DAG1 protein in bexarotene treated and untreated differentiating myoblasts, respectively, as revealed by the quantitative Western blot analysis (Figures 4A,B). More importantly, there was an over 50% reduction of myosin heavy chain expression in differentiating DAG1 knockdown myoblasts compared to the shRNA control myoblasts (Figures 4A,C), suggesting a functional role for DAG1 in myogenic differentiation.

Next, we used quantitative microscopic analysis to examine the impact of DAG1 knockdown on myoblast differentiation and fusion. The *Dag1* shRNA and non-silencing shRNA myoblasts were differentiated for 4 days and then subjected to quantitative microscopic analysis of the myosin heavy chain and nuclei staining. As shown in Figures 4D–F, there was nearly 50% reduction in the rate of myoblast differentiation and fusion due to the knockdown of endogenous DAG1 compared to that in shRNA control myoblasts, demonstrating the importance of DAG1 protein for myoblast development. Taken together, our data suggest that DAG1 may play a functional role in the process of early myoblast differentiation and fusion.

## Discussion

The correct expression of *Dag1* gene is critical for muscle integrity, particularly for the cycles of contraction and relaxation (Whitmore and Morgan, 2014). Through comprehensive omics analyses and molecular characterization, we have determined that *Dag1* gene expression is responsive to RXR-selective signaling in early myoblast differentiation. In addition, *Dag1* is a genetic target of the muscle master regulator MyoD and residue-specific histone acetylation coincides with p300 occupancy at the *Dag1* locus. As such, the p300 function is important for rexinoid-augmented *Dag1* gene expression. Finally, dystroglycan plays a role in early myoblasts differentiation. Our data shed new light on the regulation of *Dag1* gene expression and the functional relevance of dystroglycan in myogenesis.

While dystroglycan is known for its role in connecting the extracellular matrix and cytoskeleton as a component of the DGC to maintain skeletal muscle integrity (Rader et al., 2016), the molecular mechanisms regarding the regulation of *Dag1* gene expression is less clear. We show for the first time that *Dag1* gene expression is upregulated as early as day 1 of myoblast differentiation, which responds to RXR-selective signaling. The antagonist of RXRs attenuates rexinoid-responsive *Dag1* gene expression (Figure 1) because rexinoids are ligands that activate the RXRs to modulate gene transcription. Likewise, knockdown of RXRα with gene-specific shRNA abolishes rexinoid-augmented *Dag1* gene expression (Figure 1). Therefore, rexinoids regulate *Dag1* gene expression through the activation of RXR, which may be functionally relevant to the role of dystroglycan in early myoblasts differentiation. However, shRNA knockdown of RXRα also negatively affects normal *Dag1* gene expression, signifying a role of unliganded RXRα in normal myoblast differentiation.

**FIGURE 5:**
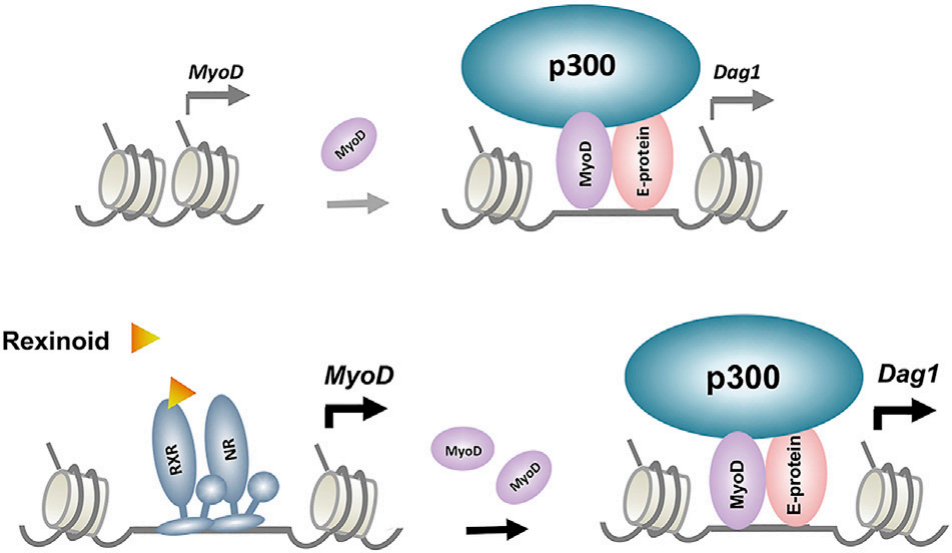
Model proposed for the regulatory events underlying p300 and MyoD-mediated rexinoid signaling in *Dag1* gene expression. RXR and its dimerization partner, nuclear receptor (NR), enhance *MyoD* expression, which in turn augments target gene expression such as *Dag1*.

While our recent RXR ChIP-seq dataset (GSM2478303-2478305) did not reveal specific RXR read signal enrichment at the *Dag1* locus, we have previously established that the muscle master regulator MyoD is a direct genomic target of RXR (Hamed et al., 2017). Therefore, the upregulation of MyoD protein level by rexinoids through the function RXR as a transcription factor may have a positive impact indirectly on *Dag1* gene expression (Figure 5). As such, we sort to explore the publicly available MyoD ChIP-seq data obtained from C2C12 proliferating and differentiating myoblasts (Yue et al., 2014). The ChIP-seq data allowed us to narrow down two regions of interest within the *Dag1* locus, both exhibiting specific enrichments of MyoD read signals at Day 1 of myoblast differentiation. One is the previously identified *Dag1* promoter near the TSS (Rettino et al., 2009), and the other is an intergenic region approximately 10 kb upstream of the *Dag1* TSS.

Enhancers are important for regulating tissue-specific gene expression and are often found at large distal regions of the muscle genes they impact. Functional enhancers are also largely associated with residue-specific histone acetylation along with the p300 occupancy (Karmodiya et al., 2012; Khilji et al., 2018). Thus, a highly accurate signature for predicting muscle-specific enhancer activity is the occupancy of p300 (Visel et al., 2009). Based on our recent omics datasets on p300 and histone acetylation, at day 1 of differentiation, the identified upstream MyoD binding site becomes a region of p300 association, which is further amplified in response to rexinoid signaling (Figure 2). Furthermore, the enrichment read signals for H4K8, H3K9, and H3K18 acetylation parallels those of the p300 occupancy (Figure 2). Importantly, this upstream MyoD binding site marked by p300 occupancy and histone acetylation is highly conserved across vertebrate species, including humans, similar to the *Dag1* promoter and the coding sequences (Figure 2). As histone modifications in promoters or intergenic enhancer regions are the most conserved, this upstream region may be highly relevant to *Dag1* gene expression.

Interestingly, this MyoD binding site is classified as a poised enhancer by our established chromatin state model in committed myoblasts as it is marked by H3K4me1 but not active histone acetylation. Quantitative ChIP analysis indeed demonstrates that this upstream region, about 10 Kb upstream of *Dag1* TSS, is p300-dependent and responds to RXR-selective signaling in early myoblast differentiation (Figure 2). Importantly, MyoD binding to this site correlates with the profile of p300 occupancy (Figure 2). Taken together, our data suggest that p300 is involved in *Dag1* gene expression, which may be concerted by MyoD that plays a specific role in recruiting p300 to activate poised enhancers in addition to the *Dag1* promoter, in early myogenic differentiation (Khilji et al., 2018).

To assess the functional requirement of p300 in *Dag1* gene expression, we examined the expression of *Dag1* in an established myoblast model with p300 knocked down for a loss of function study (Chen et al., 2015). Lack of endogenous p300 in the myoblasts leads to the significant reduction of *Dag1* gene expression in differentiating myoblasts (Figure 3). Additionally, RXR-selective signaling cannot augment D*ag1* gene expression in the absence of p300 (Figure 3). To ascertain that the reduction of DAG1 level is not due to posttranslational modification of the protein, we also assessed the mRNA level of *Dag1* in corresponding conditions. Similarly, p300 knockdown decreases the *Dag1* mRNA levels in differentiating myoblasts (Figure 3). In addition, p300 knockdown attenuates the positive effect of rexinoid signaling on *Dag1* gene expression. Thus, our data suggest that *Dag1* gene expression is, at least in part, under the control of p300 when recruited by MyoD in early myogenic differentiation (Figure 5). Intriguingly, upstream *Dag1* TSS, RXR-selective signaling is particularly coupled with the H3K18ac but not H3K27ac identified as a TSS-preferred mark (Rajagopal et al., 2014). Moreover, p300 occupancy coincides with the enrichment read signals of H4K8ac and H3K9ac upstream *Dag1* TSS, specifically in response to RXR-selective signaling. As such, our study provides additional molecular insights into how residue-specific histone acetylation may be related to *Dag1* locus activation and consequently gene transcription.

Comprehensive RNA-seq analysis leads us to dystroglycan as a rexinoid-responsive gene in early myogenic differentiation. As part of the DGC, dystroglycan has been known for its role in connecting the extracellular matrix and cytoskeleton to maintain skeletal muscle integrity (Rader et al., 2016). Interestingly, we found that dystroglycan is expressed early in normal myoblasts differentiation and is important for rexinoid-enhanced myogenesis (Figure 4). Therefore, our finding reveals a potential new role for dystroglycan in myogenic signaling and suggests that myogenic differentiation and sarcolemma integrity are intimately connected in the homeostasis of skeletal muscles.

## Data Availability

The omics cited in this study have been deposited in the National Center for Biotechnology Information Gene Expression Omnibus (GEO) database, assigned as GSE94560 (RNA-seq data) and GSE109636 (p300 ChIP-seq data).
