# Supplementary figures and images for: Regulation of Dystroglycan Gene Expression in Early Myoblast Differentiation
Source: Front Cell Dev Biol. 2022 Mar 7;10:818701. doi: 10.3389/fcell.2022.818701 (PMC8940196; doi:10.3389/fcell.2022.818701)

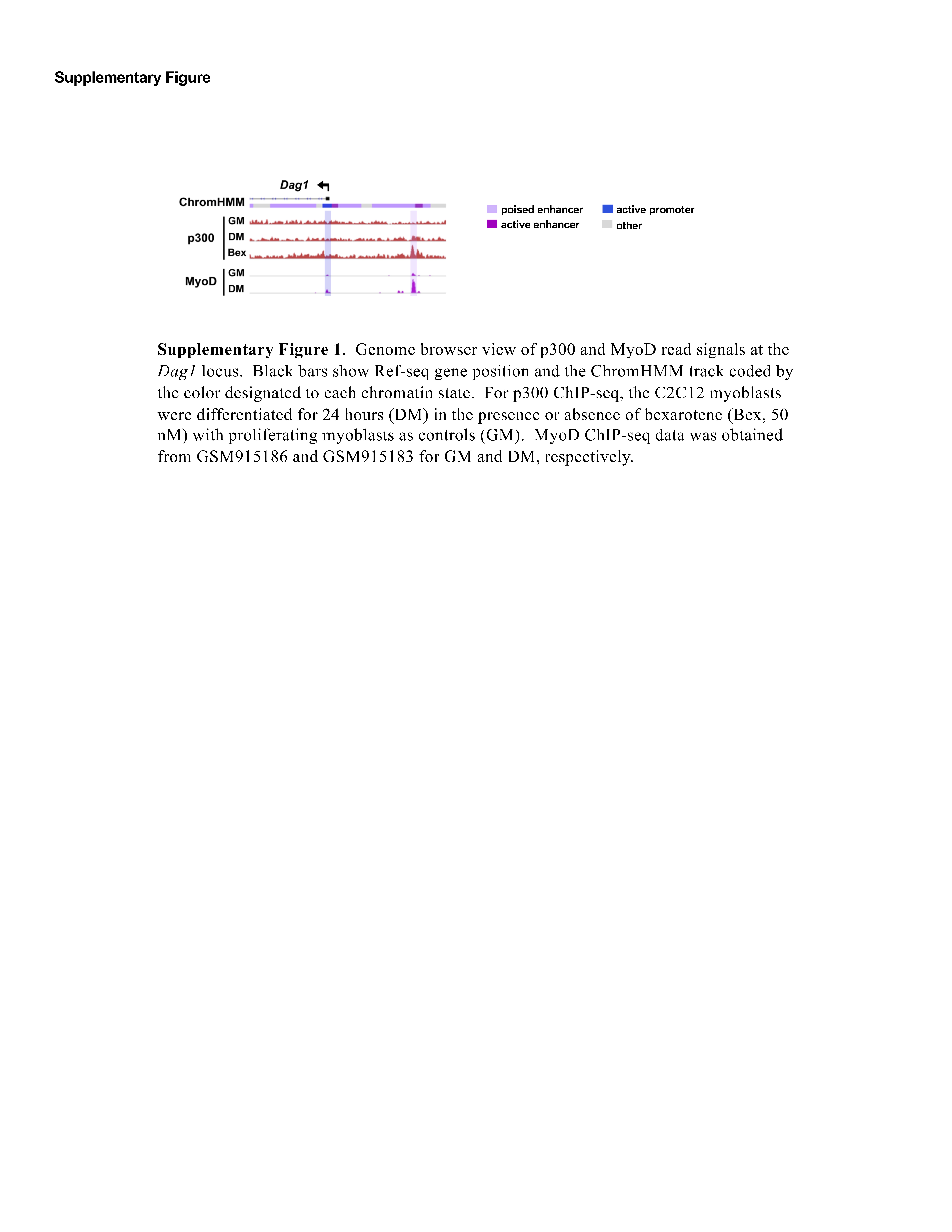

Supplement: Supplementary file 1 [file Image1.JPEG]
